# A highly stable blood meal alternative for rearing *Aedes* and *Anopheles* mosquitoes

**DOI:** 10.1371/journal.pntd.0006142

**Published:** 2017-12-29

**Authors:** Ted Baughman, Chelsea Peterson, Corrie Ortega, Sarah R. Preston, Christopher Paton, Jessica Williams, Amy Guy, Gavin Omodei, Brian Johnson, Helen Williams, Scott L. O’Neill, Scott A. Ritchie, Stephen L. Dobson, Damian Madan

**Affiliations:** 1 Intellectual Ventures Laboratory and Global Good, Bellevue, Washington, United States of America; 2 Department of Entomology, University of Kentucky, Lexington, Kentucky, United States of America; 3 Australian Institute of Tropical Health and Medicine, James Cook University, Cairns, Australia; 4 Liverpool Insect Testing Establishment, Liverpool School of Tropical Medicine, Liverpool, United Kingdom; 5 Institute of Vector Borne Disease, Monash University, Clayton, Victoria, Australia; 6 MosquitoMate, Inc., Lexington, Kentucky, United States of America; University of Texas Medical Branch, UNITED STATES

## Abstract

We investigated alternatives to whole blood for blood feeding of mosquitoes with a focus on improved stability and compatibility with mass rearing programs. In contrast to whole blood, an artificial blood diet of ATP-supplemented plasma was effective in maintaining mosquito populations and was compatible with storage for extended periods refrigerated, frozen, and as a lyophilized powder. The plasma ATP diet supported rearing of both *Anopheles* and *Aedes* mosquitoes. It was also effective in rearing *Wolbachia*-infected *Aedes* mosquitoes, suggesting compatibility with vector control efforts.

## Author summary

Mosquito-transmitted diseases pose a serious burden to global health. Several mosquito control strategies release large numbers of modified mosquitoes into disease-endemic areas to reduce transmission. Large-scale mosquito rearing operations are necessary to support these approaches, and blood feeding of female mosquitoes is central to the success of these operations. Blood feeding is challenging, especially in the resource-poor environments where rearing for controlled releases can occur. Feeding on live humans or animals has notable logistical, ethical, economic, and safety challenges. In many circumstances, feeding blood in an artificial feeder is an attractive alternative; however, rapid degradation limits the suitability of stored blood as a reliable mosquito diet to one week. The logistics associated with sourcing blood on a weekly basis can be prohibitive. Here, we demonstrate that a simple diet consisting of plasma and ATP effectively supports propagation of *Aedes*, *Anopheles*, and *Wolbachia-*infected *Aedes* mosquitoes. In contrast to whole blood, the diet is effective after extended storage under refrigerated, frozen, and lyophilized conditions. We expect this formulation to prove useful for research and control operations with blood feeding difficulties.

## Introduction

Mosquitoes transmit many life-threatening diseases such as malaria, dengue virus (DENV), and Zika virus (ZIKV), which cause over one billion infections and one million deaths annually. Increased global mobility has driven the rapid spread of many vector-borne diseases, such as ZIKV, into countries where they were previously nonexistent [1]. In the absence of effective vaccines or chemotherapies, vector control is the most direct option to limit the spread of mosquito-borne diseases.

Mosquitoes are purposefully reared for research and intervention. Female mosquitoes require a blood meal to complete oogenesis; therefore, blood feeding is a necessary step in any sustained rearing process that includes egg production. A large amount of research focused on mosquitoes and mosquito-borne diseases relies on a consistent supply of lab-reared mosquitoes. In addition, research is also performed on wild mosquito populations, such as efforts that monitor the spread of insecticide resistance [2]. In many cases, these captured mosquito populations must be expanded to produce sufficient numbers for experimentation.

Aside from research and monitoring, mosquitoes are also reared for applied purposes, such as the production of attenuated pathogens for vaccine development [3]. Similarly, mosquito rearing is essential for a variety of vector-borne disease control methods that release large numbers of modified mosquitoes. In one approach, irradiated, sterile male mosquitoes are released with the goal of collapsing mosquito populations by forming inviable offspring with endemic female mosquitoes [4].

Recent efforts have focused on additional methods to collapse or transfect wild mosquito populations to reduce mosquito-borne disease [5]. For instance, genetically-modified mosquitoes that produce defective offspring under natural conditions is a promising approach for disease control. This method is currently used for the control of DENV and ZIKV-carrying *Aedes* mosquitoes in several South American countries [6–10]. Alternatively, release of mosquitoes inoculated with the endosymbiont *Wolbachia—*which can impose conditional sterility through cytoplasmic incompatibility—has successfully suppressed *Aedes* populations [11]. This approach is currently being evaluated within the United States for control of West Nile Virus and ZIKV.

An alternative strategy using *Wolbachia*-infected mosquitoes aims to transfect local *Ae*. *aegypti* populations with the virus-blocking endosymbiont *Wolbachia pipientis* to reduce the risk of DENV and ZIKV transmission. DENV and ZIKV reproduction is significantly diminished in mosquitoes infected with *w*Mel and *w*MelPop *Wolbachia*, suggesting that these mosquitoes have decreased vector competence [12,13]. This trait, coupled with the ability of these infected mosquitoes to replace wild mosquito populations, has led to the release of *Wolbachia*-infected mosquitoes in five countries [14–16].

Strategies to transfect or suppress mosquito populations require the production of large numbers of mosquitoes for releases and thus require a means of blood feeding mosquitoes at scale. Currently, three major approaches are used to blood feed female mosquitoes in the laboratory: feeding on live animals, human volunteers, and whole blood via a glass membrane feeder. Maintaining animals for mosquito feeding suffers from high costs, special permitting, Institutional Animal Care and Use Committee (IACUC) review, and the need for trained technicians and veterinary caregivers. In resource-limited settings, acquisition and maintenance of high-quality animal populations is often difficult. Compounding the challenge, repeatedly feeding mosquitoes from the same animal generates immunity against the host animal, which may reduce mosquito survivorship and reproduction [17,18].

Use of human volunteers as blood sources is similarly challenging. Volunteers can be reluctant, and those that do enroll are at risk for developing adverse reactions to mosquito bites. Particularly for release programs, it is imperative that the host blood be pathogen-free. While methods are available to determine if volunteers are healthy, no test is 100% sensitive.

Finally, feeding blood in artificial feeders is challenging. Blood can be difficult to source, and it must be both safe for personnel to handle and appropriate for rearing mosquitoes. Shipping blood can incur import costs, varying regulations, and delays. The short self-life of blood requires sourcing new material on a near weekly basis. Collectively, these factors make the use of stored blood untenable for many operations. Innovations to extend blood’s short shelf life without affecting its ability to function as a mosquito diet would make rearing using an artificial feeder a more attractive option because it would enable stockpiling.

To address the limitations posed by blood feeding, we investigated alternative stable diets to support *Aedes* and *Anopheles* mosquito reproduction. We sought to identify a formulation that both promotes captive mosquito population expansion and is more stable than whole blood, characteristics that are desirable for rearing mosquitoes in resource-limited situations.

We found that plasma supplemented with ATP serves as a stable diet for *Aedes* and *Anopheles* mosquitoes. Our findings suggest that the plasma ATP diet could be an effective tool for mosquito propagation for mosquito control efforts.

## Materials and methods

### Reagents

Unless otherwise indicated, the reagents, kits and materials used in this study were purchased from Sigma (St. Louise, MO).

### Rearing

*Aedes aegypti* and *Anopheles gambiae* mosquito colonies were reared according to previously published protocols [19,20].

### ATP

A buffered stock of 100 mM ATP was made by adding 1.102 g of ATP to 3.3 mL of at 4°C deionized water. 2.9 mL of 1 M NaOH was added to the solution with continuous mixing to maintain pH below pH 7.0 and then adjust to pH was 7.4 with 0.1 M NaOH. Cold 0.1 M sodium phosphate buffer (pH 7.4) was added to a final 20 mL volume with aliquots stored at -20°C. 0.1 M Na/K buffer was prepared by mixing ~1 vol. 0.1 M KH_2_PO_4_ with ~4 vol. 0.1 M Na_2_HPO_4_ until pH 7.4 was achieved. Unless otherwise indicated, all formulations that included ATP were first warmed to 37°C in a recirculating water bath. Then, ATP was added to a 1 mM concentration 10 min prior to feed initiation.

### Fecundity measurements

#### Uninfected *Ae*. *aegypti*

Whole blood with sodium heparin was purchased from Bioreclamation IVT (Hicksville, NY) and unless otherwise indicated held at 4°C until further use. Within three days of receipt, blood aliquots were separated in RBC, buffy coat, and plasma by centrifugation at 4000XG at 4°C for 10 minutes.

Plasma was transferred to a sterile container and stored at 4°C until further use. RBC was prepared by removing both plasma and buffy coat fractions. Separated then fully reconstituted blood and formulations utilizing varying volumetric ratios of RBC and plasma were recombined by end-over-end mixing. Lyophilized human plasma (MyBioSource, Inc.; cat. MBS173286) was reconstituted with water. Commercially available frozen, pooled human plasma was purchased from BioreclamationIVT (cat. HMWBNAHP).

Approximately 100 female ≥ 5 day-old adult mosquitoes and 30 males were housed in 0.3 m^3^ cages to ensure insemination. Mosquitoes were fed using Hemotek or Lillie glass membrane feeders as previously described [19]. Mosquito engorgement was visually determined immediately post feeding. Cohorts of female mosquitoes were fed test diets, and the palatability of the diets was determined by measuring the percent of females that were visibly engorged.

**Fig 1:**
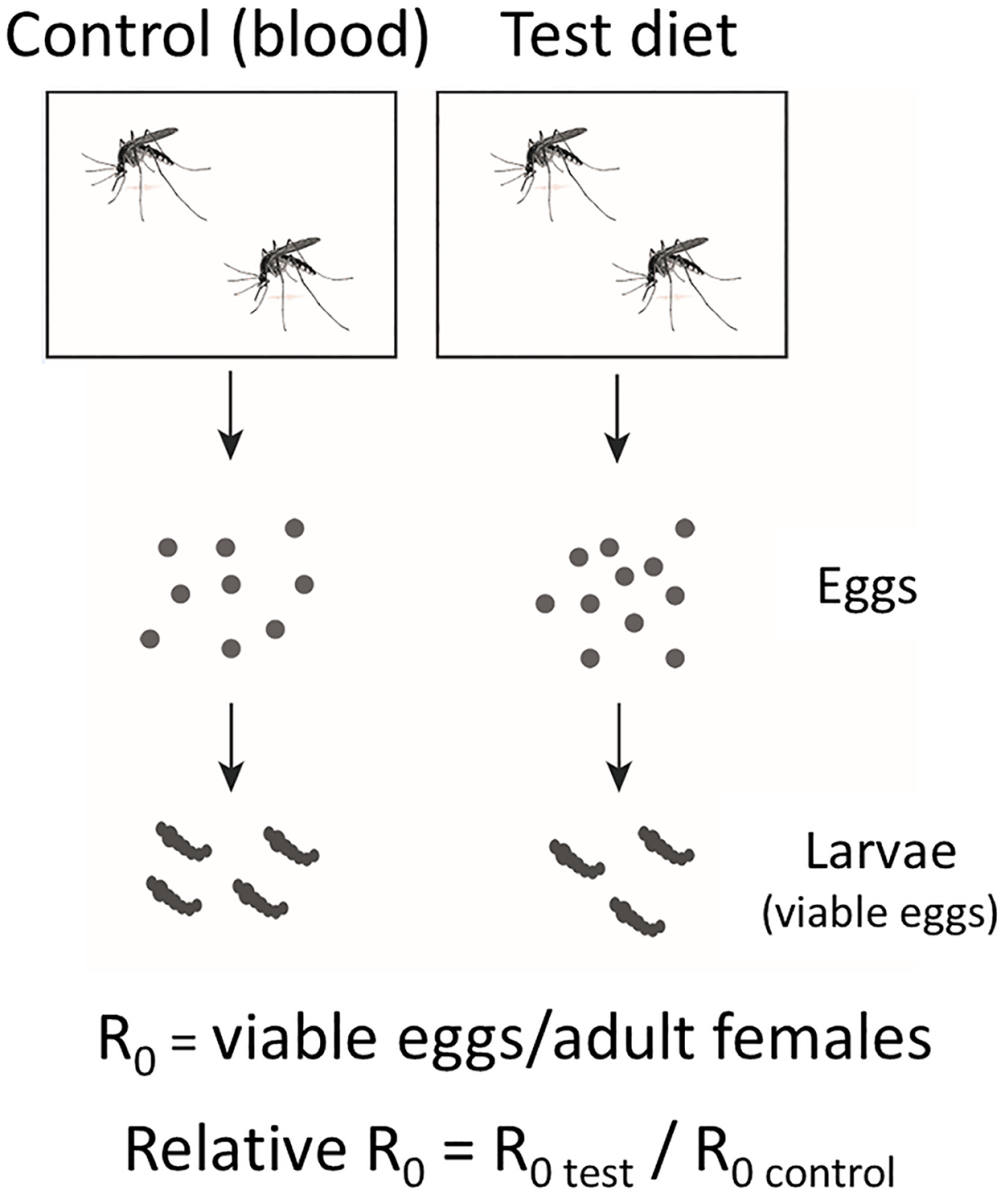
Relative R_0_ measures reproductive potential. The reproductive potential (R_0_) was determined by quantifying the number of viable eggs generated by the total number of female adults. Relative R_0_ was determined by comparing the R_0_ of test diet to blood fed controls.

Fecundity was assessed by either determining the average number of larvae produced by the total number of female mosquito offered the diet or by counting the number of hatched eggs. We refer to this ratio as R_0_ (Fig 1). To normalize results, all experiments included a fresh human blood sample control delivered via artificial feeder. We divided the R_0_ of the test diet by R_0_ of the fresh human blood control and refer to this term as the relative R_0_ (RR_0_) (Fig 1).

#### *Wolbachia-*infected *Ae*. *aegypti*

Trials involved Cairns *Ae*. *aegypti* infected with *w*Mel *Wolbachia*, F2-6 with ten replicates per treatment. Commercial plasma and less than one-week-old, discarded Red Cross whole blood were tested for the presence of key pathogens as per Red Cross protocol. Citrate Phosphate Dextrose was the anticoagulant in the whole blood samples. Mosquitoes starved for approximately 24 hours were offered blood or plasma mixtures using a heated water jacket glass feeder fitted with a collagen membrane for 60 min. Each cage was provided with 50% honey water and maintained at 70–80% RH at 26°C. Three days after feeding, two 200 mL plastic containers with red flannelette cloth ringing the inner wall and100 ml of deionized water were placed into each cage for oviposition. After 3 days, the red flannelette cloth was removed and eggs counted using a stereomicroscope. Three random sections of the egg cloth were then incubated for 3+ days at high humidity at 26°C, and then immersed in dilute yeast solution to induce hatching. Hatched first instar larvae and eggs were counted 24hr post hatch and hatch rate quantified.

#### *An*. *gambiae*

Two cages of 60 three to five day old female *An*. *gambiae* (Kisumu strain) mosquitoes were starved for six to twelve hours. Mosquitoes were from a multiyear colony maintained at Liverpool School of Tropical Medicine. One cage received the plasma ATP diet and the other cage received a blood meal using a Hemotek membrane feeder.

Post feeding females were separated into individual Falcon tubes and the number of eggs laid by each female was counted. Eggs from each female were placed in individual pots containing 200 mL distilled water. The individual pots were checked daily to determine day of hatching and the total number of first instar larvae. First instar larvae from each treatment were pooled into a tray with 1 L distilled water. The larvae not used for the larval development study were reared to adulthood and kept for the next generation.

For the larvae and pupae development study, ten first instar larvae were removed from the pool of larvae and placed in a pot with 200 mL distilled water. Groups of ten first instar larvae were removed and placed into 200 mL distilled water. Pools from generations 1, 2, and 3 contained 88, 100, and 100 first instar larvae, respectively. Emerged adults were transferred to the next generation adult cages.

Feed two consisted of 35 females for each treatment. Feed three consisted of 60 fed an artificial diet and 55 fed full blood meal. Unlike the first generation study, females in generations two and three were housed in the dark and sugar was removed to encourage laying.

### ATP stability testing

Buffered ATP (100 mM) was diluted to 1 mM in human plasma or PBS precooled to 4°C, and 0.5 mL aliquots were rapidly moved to -80, -20, 4 or 37°C. At the indicated times, samples were removed and assayed using an Adenosine 5’-triphosphae (ATP) Bioluminescent Assay Kit using the manufacturer’s provided protocol. Briefly, the supplied ATP assay mix was diluted 1:100 in water then mixed in equal volumes with assay samples in Corning, white, 96 well, flat-bottomed, polystyrene microplates plates. Luminescence was immediately read on a SpectraMax M3 Microplate reader (Molecular Devices, Sunnyvale, CA).

### Statistical analyses

All data were analyzed in GraphPad. R_0_ values of blood and fresh plasma ATP were analyzed by unpaired t test. Relative R_0_ values of blood and plasma stored at 4°C over time were analyzed for correlation by Spearman’s rank order test. Relative R_0_ values of fresh plasma ATP, premixed plasma ATP stored at -20°C for two weeks, and plasma and ATP stored separately at -20°C for two weeks then mixed prior to feeding were analyzed by ANOVA.

## Results

### The reproductive potential of *Ae*. *aegypti* female mosquitoes is maintained with a plasma-based diet

Blood is readily fractionated into red blood cell (RBC) pellet, buffy coat, and plasma by centrifugation. We hypothesized that these fractions both contribute disproportionately to mosquito engorgement and vitellogenesis and degrade at different rates.

We partitioned blood and recombined the components in various arrangements. Experiments compared whole, unseparated blood; RBCs combined with plasma only; and separated then fully reconstituted blood. All three experimental groups performed similarly when fed to mosquitoes (data not shown). Next, we assessed the roles of RBC and plasma by varying the volumetric ratios to produce different hematocrits in test diets. A high RBC to plasma diet (1:5) demonstrated the same RR_0_ as the high plasma to RBC diet (5:1) despite large difference in engorgement rates and protein levels (S1 Fig).

**Fig 2:**
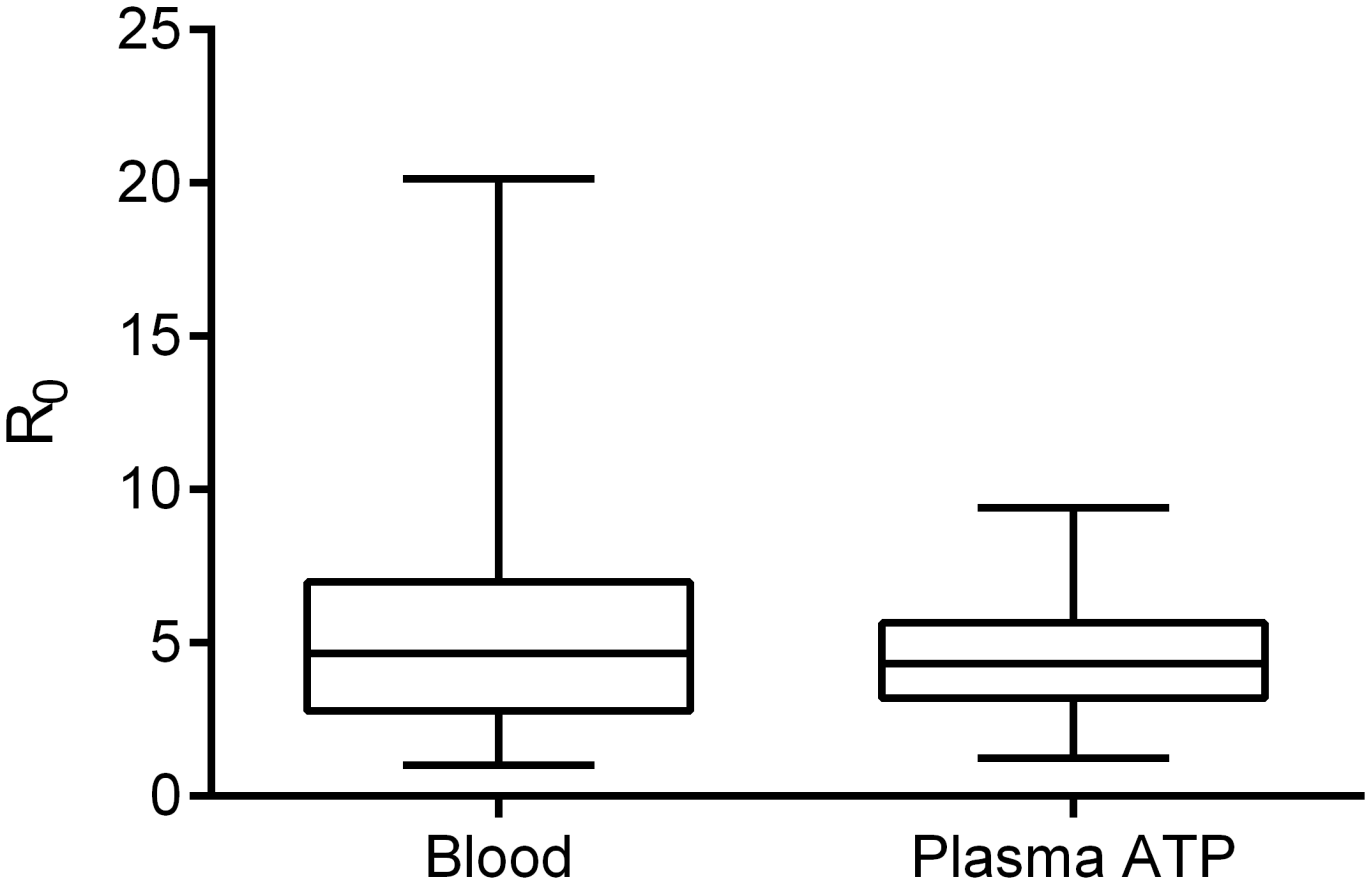
A plasma ATP diet is effective for *Ae*. *aegypti* mosquitoes. Fresh blood or plasma ATP was fed to uninfected *Ae*. *aegypti* mosquitoes via artificial feeder. R_0_ measurements are shown (N = 23 fecundity measurements for each feeding group). No significant differences were present between data sets (unpaired t test).

Guided by previous studies that found ATP is a phagostimulant, a 9:1 (plasma:RBC) mixture was spiked with buffered ATP and compared to the unsupplemented mixture [21–26]. ATP increased mosquito engorgement and RR_0_ 2.3-fold (S1C and S1D Fig). Buffered ATP was then spiked into plasma alone (no RBCs). Female *Ae*. *aegypti* fed on the plasma ATP diet displayed a reproductive potential that did not significantly differ from the blood fed control mosquitoes (Fig 2**,** *P* = 0.9730)**,** demonstrating that plasma alone is nutritionally sufficient to support vitellogenesis and could replace whole blood feeding for mosquito propagation.

### Plasma is more stable than whole blood

**Fig 3:**
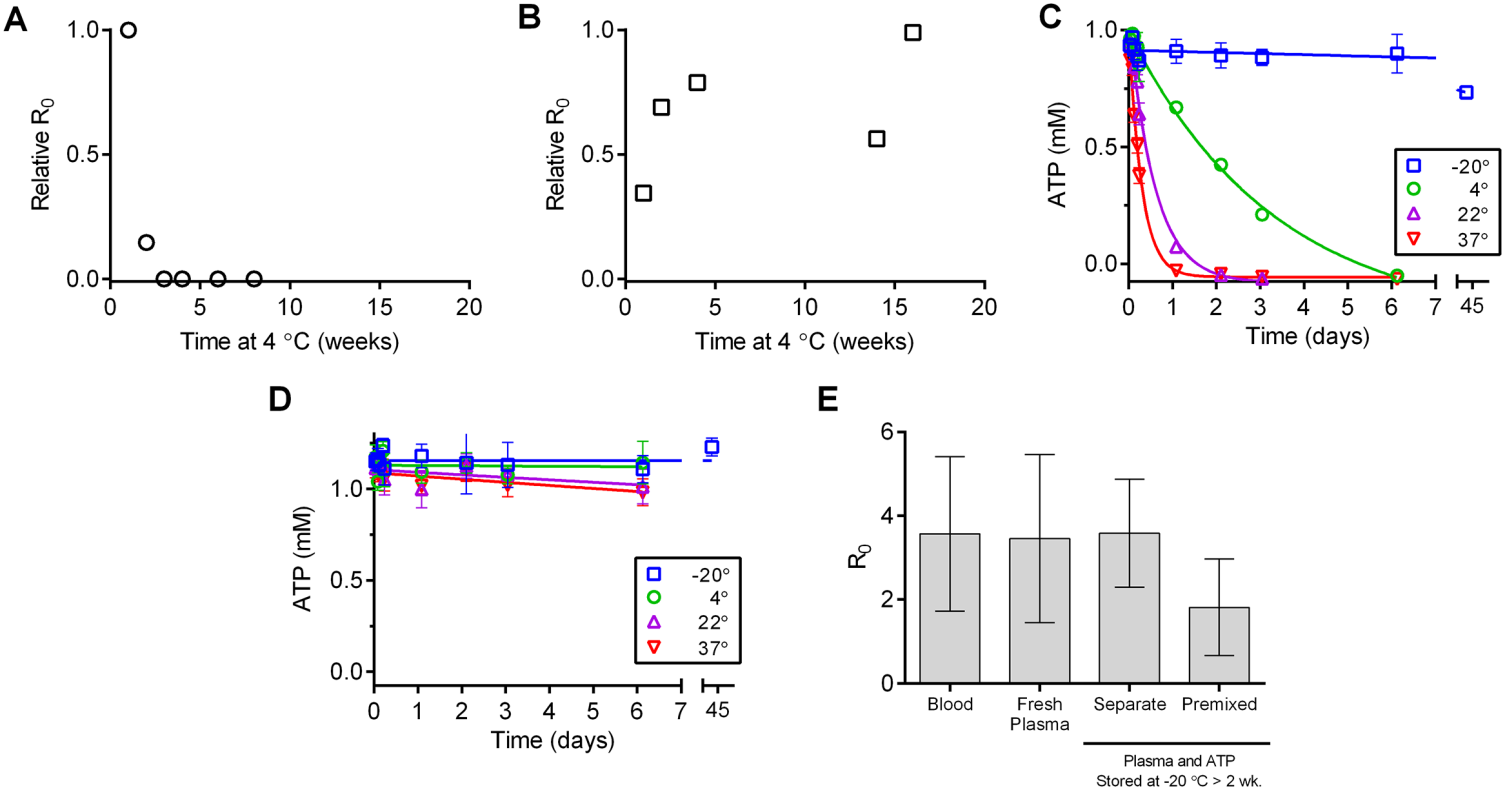
Plasma is more stable than whole blood. **A and B.** Blood **(A)** and plasma **(B)** were incubated at 4°C. Periodically, samples were fed to uninfected *Ae*. *aegypti* mosquitoes. Plasma samples were supplemented with freshly frozen ATP prior to feeding. Relative R_0_ measurements are shown. **C. and D.** Plasma **(C)** and PBS **(D)** were spiked with ATP and incubated at various temperatures. ATP concentration was measured at indicated time points (N = 3 fecundity measurements for each feeding group), error bars represent standard deviation (SD)). **E.** Plasma was incubated at -20°C alone (separate) or supplemented with ATP (premixed) for two weeks. Samples were thawed, ATP was added to the separate sample, and both samples and fresh plasma ATP (fresh) were fed to uninfected *Ae*. *aegypti* mosquitoes. Relative R_0_ measurements are shown (N = 3). No significant differences were present among data sets (ANOVA).

To assess the stability of the plasma ATP diet we stored blood and plasma stored at 4°C for up to 16 weeks. These stocks were periodically sampled and fed to mosquitoes to assess loss of diet performance. All plasma samples were supplemented with fresh, buffered ATP immediately prior to feeding. Blood performance decreased sharply after two weeks (Fig 3A; *P* < 0.0001). Encouragingly, the plasma ATP RR_0_ did not decrease over the 16 week period (Fig 3B; *P* = 0.2333).

ATP degradation was considerably accelerated in the presence of plasma, and the rate of degradation was temperature dependent (Fig 3C and 3D). However, ATP-supplemented plasma stored frozen for over two weeks did not display a significantly lower RR_0_ than when the components were stored under the same conditions but in separate vials then reconstituted immediately prior to feeding (Fig 3E; *P* = 0.4374; *F* = 0.9650).

### Commercially available human plasma and non-human plasma sustain mosquito reproduction

**Fig 4:**
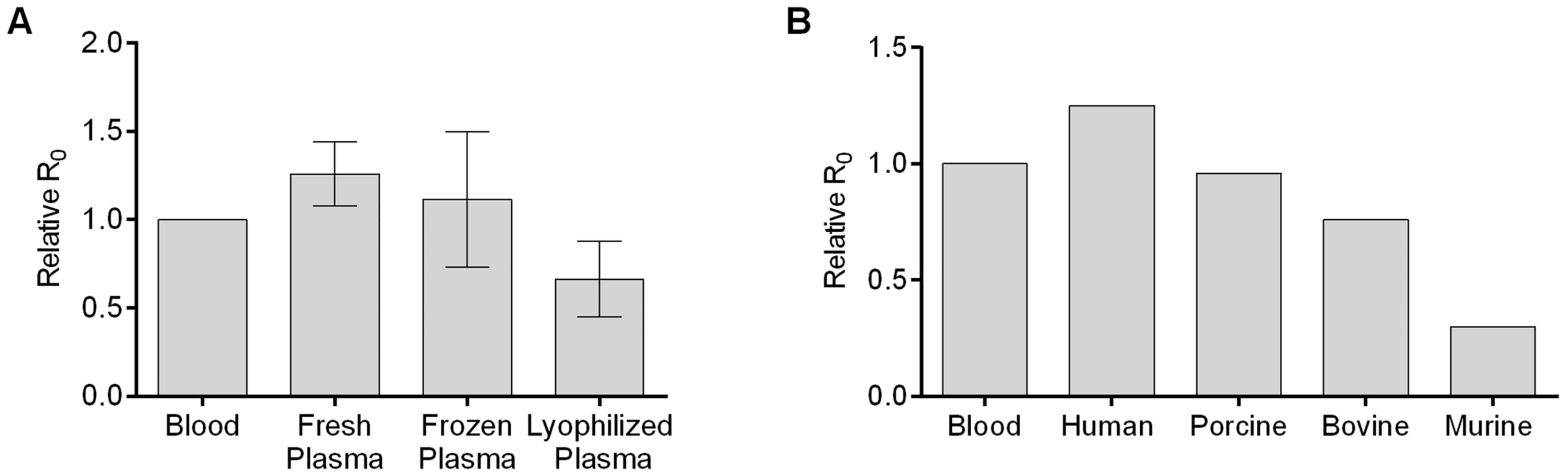
A plasma ATP diet remains effective when commercially-sourced frozen, lyophilized, or nonhuman plasma sources are used. **A.** Commercially-sourced frozen or reconstituted lyophilized pooled human plasma were supplemented with ATP and fed to *Ae*. *aegypti* mosquitoes. Relative R_0_ measurements are shown. (N = 3 fecundity measurements for each feeding group, error bars represent SD). **B.** Feeding experiments in **A.** were repeated with commercially-sourced frozen animal plasma. Data are representative of two independent experiments.

We investigated alternative plasma sources that might prove easier and or cheaper to acquire than freshly separated plasma from whole, human blood. Commercially-sourced, frozen plasma showed variable performance but sustained mosquito reproduction similar to whole blood and fresh plasma ATP diets. In contrast, reconstituted lyophilized plasma (reconstituted by end-over-end mixing with distilled water) showed inferior fecundity, with an RR_0_ of 0.5 (Fig 4A). This finding may be due to inhomogeneity in the reconstituted solutions, which typically remained turbid despite extended mixing. Although lyophilized plasma did not perform as well as frozen plasma, alternative reconstitution protocols may address these shortcomings and enable rearing in more challenging locations.

In addition to testing different sources of human plasma, we investigated the performance of several commonly available non-human plasmas: porcine, bovine and murine. We supplemented each plasma with ATP and compared to fresh human plasma and blood fed controls. The RR_0_ was 0.9, 0.75, and 0.30, for porcine, bovine and murine plasma, respectively (Fig 4B).

### The plasma ATP diet supports the propagation of *Wolbachia-*infected *Ae*. *aegypti*

**Fig 5:**
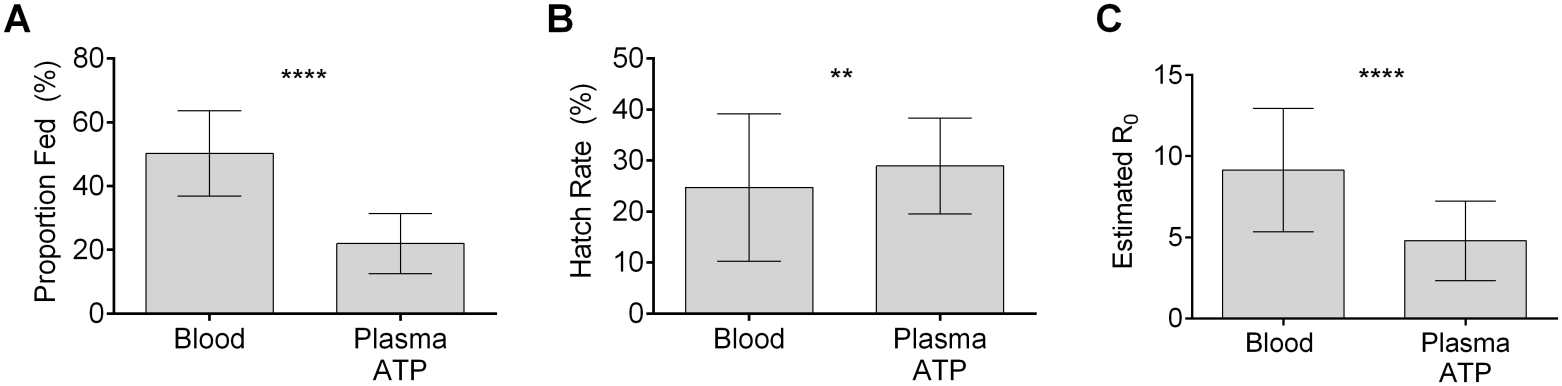
A plasma ATP diet is effective for *Wolbachia-*infected mosquitoes. **(A-C)** *wMel*-infected *Ae*. *aegypti* mosquitoes were fed the plasma ATP diet. Feeding rates (**A**), hatch rates (**B**), and estimated reproductive potential (**C**) are shown. **** denotes p < 0.0001, ** denotes p< 0.01, (N = 10 fecundity measurement for each feeding group. Error bars represent SD).

A stable human blood alternative could enable operations that rear and release *Wolbachia*-infected mosquitoes, particularly in resource-limited situations. We compared the efficacy of whole human blood and the plasma ATP diet to support *w*Mel-infected *Ae*. *aegypti*. Encouragingly, while the plasma ATP diet displayed reduced fecundity as compared to whole blood, it did produce viable offspring with an estimated R_0_ of 4.8±0.5 (Fig 5), suggesting that this diet is capable of supporting population expansion of *Wolbachia*-infected mosquitoes.

### The plasma ATP diet supports multigenerational propagation of *An*. *gambiae*

Overall, our data show that mosquitoes offered a single meal of human plasma supplemented with ATP or whole human blood produce similar numbers of viable eggs. While suggestive, these studies alone do not determine if these eggs develop into healthy adult mosquitoes or whether the plasma ATP diet is capable of supporting multigenerational rearing.

**Fig 6:**
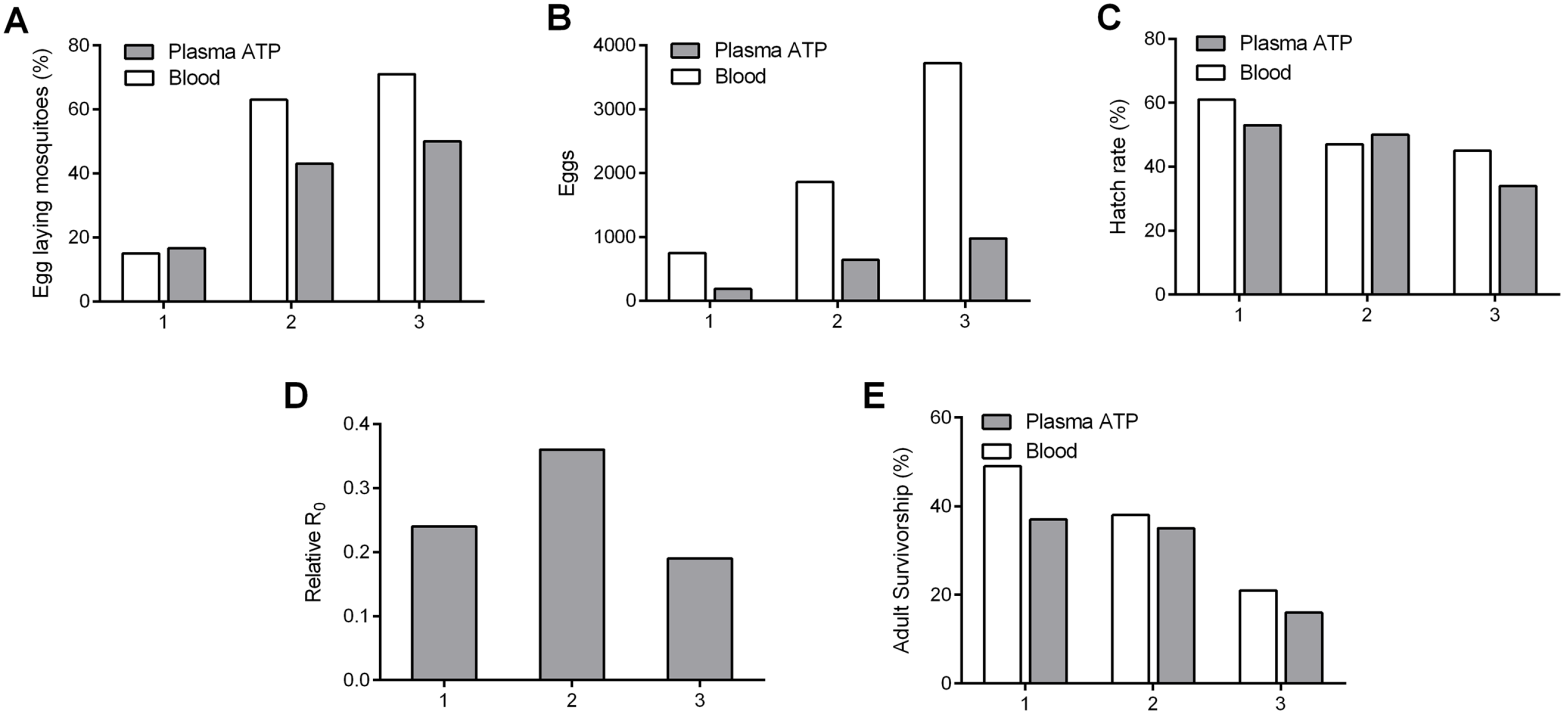
A plasma ATP diet sustains multiple mosquito generations. *An*. *gambiae* were fed the plasma ATP diet or blood via artificial feeder for three generations. The percent of laying females (**A**), number of eggs laid (**B**), hatch rate (**C**), relative R_0_ (**D**) and adult survivorship (**E**) are shown.

We compared viability of plasma-ATP-fed *An*. *gambiae* mosquitoes to blood-fed controls for three generations. Both blood and plasma ATP diets supported propagation over the three generations (Fig 6). In contrast to *Ae*. *aegypti* mosquitoes, fecundity of plasma-ATP-fed *An*. *gambiae* mosquitoes was notably reduced compared to blood-fed cohorts (Figs 2 and 6). For both diets, generational differences were seen in different propagation metrics, suggesting that factors other than diet affected R_0_. Within each generation the two diets resulted in similar hatch and adult survivorship rates. The decreased fecundity of the plasma ATP diet was largely influenced by reduced numbers of egg-laying mosquitoes in generations two and three and reduced overall egg numbers in plasma-fed mosquitoes in all generations (Fig 6).

## Discussion

Mosquito rearing represents a significant logistical commitment in both ongoing vector research and vector control strategies. This work focused on reducing the logistical burdens associated with mosquito rearing by improving the quality, stability, and performance of mosquito foods, particularly with respect to field deployment.

A variety of artificial mosquito diets have been developed from *de novo* methods and materials [27–29]. In our hands, we found that these alternatives showed poor reproducibility, extreme species dependence, and high complexity. Rather than attempt to synthetically recapitulate a feeding relationship that has evolved over millennia, we instead chose to focus on the essential elements of the mosquito blood meal.

We investigated different blood components’ contributions to gorging and fecundity. Our findings are in accordance with previous reports that showed ATP is an important driver for mosquito engorgement. Because ATP is disproportionately associated with RBCs, plasma alone is not consumed readily by mosquitoes [26]. However, feeding is stimulated with ATP supplemented plasma.

Interesting, fecundity was not affected when we fed mosquitoes plasma ATP instead of whole blood. This finding implies that protein content alone is not the sole driver of mosquito fecundity, since RBCs are considerably more protein rich than plasma. However, RBC protein is dominated by hemoglobin, and human hemoglobin is deficient in isoleucine, an essential component of the mosquito blood meal [30,31]. With respect to mosquito feeding, a smaller quantity of protein with a balanced amino acid content appears equivalent to a larger quantity of isoleucine-deficient protein.

Blood is perishable and mosquito-based research and vector control efforts could benefit from maintaining mosquitoes on a stable artificial blood diet. Refrigerated plasma was effective for at least 16 weeks (our oldest sample tested). This was in marked contrast with refrigerated whole blood, which did not support mosquito feeding after two weeks. Moreover, both human and non-human plasma are commercially accessible and support rearing.

In addition to assessing plasma stability, we assessed storage conditions for ATP since it is subject to degradation. When stored separately in pH 7.4 buffer, ATP is stable for more than one month and does not require freezer storage. The simplicity of the plasma ATP diet, ease of storage, and increased stability make it an attractive alternative to blood feeding, especially since this the diet supported mosquito propagation for multiple generations. Our experiments did show decreased fecundity of the plasma ATP diet over multiple generations. However, the fecundity of the whole blood control also decreased by similar magnitudes, highlighting the complexity of mosquito rearing.

*Ae*. *aegypti* mass propagation is needed for test trials to collapse susceptible, wild mosquito populations and potentially reduce the prevalence of DENV and ZIKV within the United States. In our studies, *wMel*-infected mosquitoes exhibited half the reproductive potential as blood fed controls. This is an improvement over previous reports, which found that the fecundity of *Wolbachia*-infected mosquitoes was severely limited when fed on non-human blood sources, with blood from some species exhibiting R_0_ values of less than one [32]. The effect of infection or genetic modification on mosquito feeding is poorly understood, and the plasma ATP diet may need modifications to tailor it to specific mosquito strains. In the event that propagation efficiency is too low, additional work to augment or refine formulations for *wMel*-infected *Ae*. *aegypti* may be necessary. However, the plasma ATP diet should provide a simple and consistent basis for further dietary investigations.

In virtually all studies, artificial feeders deliver inferior feeding results relative to the gold standard: live feeding on human volunteers. However, the limitations of this strategy are particularly important in the context of vector management using live release strategies. In addition to the obvious problems with donor recruitment and fatigue, the sensitivity, specificity, and turnaround time in donor disease screening is considerable. For approaches that release female mosquitoes, the risk of inadvertently releasing infected mosquitoes is highly important, as even a single adverse event could erode public tolerance for further trials. In some cases, a lower rate of mosquito production from a stable and readily screened dietary formulation may be an acceptable trade-off.

This study focused on comparing the relationship between fecundity and stability of blood meal alternatives. While important, these features do not exhaust the considerations an operation would assess prior to adopting this feeding strategy. The performance of the plasma-based diet across greater than three generations was not tested, and physiological effects, such as altered fitness and genetic or phenotypic drift, may exist. Genetic drift has been documented in lab reared versus wild type mosquitoes [33–35], and could ultimately result in meaningful phenotypic variance. Evaluating long terms effects on mosquito physiology is outside of the scope of this particular and would be important for future investigation.

Furthermore, the reported results can inform the basis for future studies, which could explore augmenting the base diet with feeding additives. An investigation of stabilized ATP surrogates could add further refinement for field deployments. A more detailed investigation of lyophilization or other drying procedures such as spray drying to control lot variability could further extend the field utility of the existing diet. The existing work provides a coherent starting platform for a simple and effective diet.

## Supporting information

S1 Fig A primarily plasma-based diet supplemented with ATP supports egg development. Increasing concentrations of plasma were fed to *Ae*. *aegypti* female mosquitos. **A**. Engorgement percentage and **B**. reproductive potential as determined by RR_0_ were assessed to determine whether hematocrit was necessary. ATP was tested as an enhancing agent to increases C. engorgement and D. reproductive potential.
(TIF)
